# Neuroendocrine factors - predictors of the formation of alcohol dependence and human ecology in various ethnic populations, new approaches to therapy

**DOI:** 10.1192/j.eurpsy.2021.1893

**Published:** 2021-08-13

**Authors:** T. Shushpanova, A. Mandel, N. Bokhan, T. Novozheeva

**Affiliations:** 1 Clinical Psychoneuroimmunology And Neurobiology Lab, Mental Health Research Institute, Tomsk, Russian Federation; 2 The Department Of Addictive States, Mental Health Research Institute, Tomsk National Research Medical Center of the Russian Academy of Sciences, Tomsk, Russian Federation

## Abstract

**Introduction:**

Integrated clinical and biological approaches in the study of disorders caused by alcohol consumption in people of different ethnic groups, are important for determining effective treatment strategies.

**Objectives:**

To study the clinical and dynamic features and the role of neuroendocrine factors of the formation and course of alcohol dependence in individuals of Tuvan ethnicity.

**Methods:**

68 Russian alcoholics and 67 Tuvans alcoholics only men and 20 healthy male were monitored. Clinical assessment of the condition of patients was carried out with the traditional clinical description. Enzyme-linked immunosorbent assay kits were used to determine serum hormone levels in patients and volunteers.

**Results:**

The systematic consumption of alcoholic beverages develops among Tuvans in adulthood, in contrast to Russian men who begin to drink systematically young. Alcohol dependence in people of Tuvan nationality are formed several years later than in Russians: a symptom of loss of quantitative control over use was detected in Tuvans at 36.9±9.9 years, in Russian patients at 29,8±7.5 years; the formation of withdrawal syndrome in Tuvans occurs at the age of 37.7±8.4 years, unlike Russians, in whom the withdrawal syndrome develops on average at the age of 29.6±6.0 years. The index of the ratio cortisol/progesterone in the blood of examined Russian alcoholics is almost twice as high as the index of examined healthy donors; in patients of Tuvan ethnicity, index is almost five times higher.

**Conclusions:**

Alcohol dependence among representatives of the Tuvan ethnic group indicates a greater vulnerability to the effects of alcohol.

**Disclosure:**

No significant relationships.

